# Swedish middle school students’ psychosocial well-being during the COVID-19 pandemic: A longitudinal study

**DOI:** 10.1016/j.ssmph.2021.100942

**Published:** 2021-10-09

**Authors:** Emily G. Vira, Therése Skoog

**Affiliations:** Department of Psychology, University of Gothenburg, Gothenburg, Sweden

**Keywords:** Psychosocial well-being, COVID-19 pandemic, Longitudinal study, Sweden, Middle school students

## Abstract

Child well-being concerns amidst the COVID-19 pandemic have been reported from countries with strict lockdowns and school closures. Sweden's middle school students attended school as normal during the pandemic, but it is still unknown how their well-being has changed during the pandemic. This study aimed to assess differences in Swedish students' psychosocial well-being from before to during the pandemic. Longitudinal data (N = 849) were collected via self-report surveys across two time-points separated by approximately one year. The second data collection took place 8–9 months after the start of the pandemic in Sweden. We measured psychological adjustment, relationships to significant others and school adjustment. Findings revealed significant mean-level decreases in students' school adjustment during the pandemic. There were no meaningful mean-level decreases in students' relationships to significant others. Students' psychological adjustment showed significant but mostly negligible mean-level decreases, and no differences in emotional problems during the pandemic. The findings are explained based on students' developmental stage and the handling of the pandemic in the Swedish school context. Based on this first longitudinal study on students' wellbeing during the COVID-19 pandemic, we conclude that Swedish middle school students who continued formal schooling show mainly positive adaptations, and thus appear to be resilient during the COVID-19 pandemic.

## Highlights

• This is the first longitudinal study focusing on Swedish middle school students' psychosocial well-being during the COVID-19 pandemic.
• Significant mean-level decreases were found in students' school adjustment, quality of relationships to significant others and psychological adjustment, when comparing levels from prior to the pandemic to during the pandemic.
• Findings are explained based on students' developmental context and the context of the COVID-19 pandemic in Sweden
• Despite significant decreases in students' psychosocial well-being, students showed no differences in emotional problems, showing support for students' resilience during the COVID-19 pandemic.

## 1 Introduction

The COVID-19 pandemic has affected the globe in a multitude of ways, including increasing psychological distress in the general population, among healthcare workers (Lu et al., 2021), vulnerable populations (Otu et al., 2020), and amongst families (Spinelli et al., 2020); including effects on children's day-to-day lives. Scholars and practitioners working with children, have been concerned about the psychosocial well-being of children during the pandemic (Cui et al., 2020; Tso et al., 2020). In their review of studies published in the first part of the pandemic, Gul and Demirci (2021) reinforced this concern in concluding “The ongoing COVID-19 pandemic is causing serious mental health problems in growing children” (p.27). To aid in controlling the spread of infection, many countries have moved formal schooling for children online. In another review of studies performed predominantly during the pandemic, school closures were correlated with harms on children's mental well-being (Viner et al., 2021). Assessing the immediate effects of the pandemic in China, Xie et al. (2020) reported that 22% of children in grades 2–6 showed depressive symptoms when restricted to home-confinement. Such negative effects were also found in Europe amongst Spanish, Italian, and Portuguese children, with parents reporting an increase in other internalizing symptoms (e.g., anxiety and loneliness) in comparison to before the pandemic (Francisco et al., 2020). A UK-based longitudinal survey on children and adolescents' mental health reported increases in mental health problems when lockdowns were most strict and school closures ensued (Cresswell et al., 2021).

Although these findings raise apprehensions about children's well-being, they should be interpreted with some caution. Even though longitudinal research is needed to assess the impact of the pandemic, only a small minority of studies have been longitudinal with pre- and mid-/post-pandemic comparisons of children's well-being. Robinson et al. (2021) reported that mental well-being decreases are typically found during the early stages of the pandemic, while mental well-being in later stages of the pandemic show a return to pre-pandemic levels. Moreover, there are few studies on children younger than 13 years. Of those studies that have included younger children, findings suggest children (7–10 years) report poorer mental well-being than preadolescents and adolescents (Ravens-Sieberer et al., 2021).

In sharp contrast to many other societies, Sweden has allowed schools up to grade six to remain open and continue formal schooling during the pandemic. Recent longitudinal findings from Sweden have found that adolescents – for whom schools have been partly closed – have experienced slight decreases in their psychosocial well-being during the first year of the pandemic (Chen et al., 2021). Still, we are unaware of longitudinal studies that examined aspects of psychosocial well-being or school adjustment for children in Sweden during the pandemic. This study's aim was to compare Swedish middle school students' (grade 4–5) psychosocial well-being before the pandemic to approximately a year into the pandemic.

RQ1 Is there a difference between Swedish students' psychological adjustment prior to and during the COVID-19 pandemic?

RQ2 Is there a difference between Swedish students' quality of their connections to significant others prior to and during the COVID-19 pandemic?

RQ3 Is there a difference between Swedish students' school adjustment prior to and during the COVID-19 pandemic?

## 2 Method

We analyzed data from children in 30 middle schools in western Sweden belonging to the longitudinal Peer Relations In School from an Ecological perspective (PRISE) project (Skoog et al., 2019). Data were collected in October 2019–January 2020 (T1) prior to Sweden's pandemic outbreak (first cases confirmed on 24/02/2020), and one year later (T2), when the weekly rate of confirmed COVID-19 cases varied from 20,000–46,000 during November 2020–February 2021. At T1, data were collected from 1006 fourth-grade students. At T2, during the pandemic, data were collected from 979 fifth-grade students. Only students who had completed questionnaires from both time-points have been included in this study (N = 849).

### 2.1 Participants

Mean age at T1 was 10 years (*SD* = .03, range 9–11 years) and 11 years at T2 (*SD* = .05, range 10–12 years). Of participants, 403 were boys, 440 girls, and 6 gender non-conforming. Overall, 93.5% were of Swedish ethnicity (determined by asking participants where they were born), 88.6% of parents were from Sweden or another Scandinavian country, while 11% of both parents were born in another country. To assess participants’ socio-economic status (SES), students were asked whether they had their own room, with 86.4% reported that they had their own room at T1 and 89.5% had their own room at T2.

### 2.2 Measures

#### 2.2.1 Psychological adjustment

##### 2.2.1.1 Sense of hope

The Children's Hope Scale (CHS; Snyder et al., 1997) had six items asking about participants' sense of hope, e.g., “I think the things I have done in the past will help me in the future”, and how they manage situations, e.g., “When I have a problem, I can come up with lots of ways to solve it”. Responses were given using a 6-point Likert scale ranging from (1) *none of the time* to (6) *all of the time* (T1, α = .82; T2, α = .86).

##### 2.2.1.2 Self-efficacy

The Children's Self-Efficacy Scale (CSES) had four items relating to participants' ability to be assertive and expressive, rated on a scale from 0 to 100 how certain they were with their capabilities to, for example, “Stand up for myself when I feel I am being treated unfairly” and “Express my opinions when other classmates disagree with me” (T1, α = .79; T2, α = .78). The measure was taken from Bandura’s (2006) guide on constructing self-efficacy scales.

##### 2.2.1.3 Self-esteem

The Single-Item Self-Esteem Scale (SISE; Robins et al., 2001) had one item: “I have high self-esteem”. Responses were measured on a five-point Likert scale ranging from (1) *not true at all* to (5) *completely true*.

##### 2.2.1.4 Emotional problems

The subscale of emotional problems from the Strengths and Difficulties Questionnaire (SDQ-sve, Lundh et al., 2008) had five questions about experiences with emotional problems, e.g., “I am often sad, depressed or ready to cry”. Items were answered on a three-point Likert scale with the values of (1) *not true*, (2) *partly true*, and (3) *completely true* (T1, α = .71; T2, α = .72).

#### 2.2.2 Connections to significant others

##### 2.2.2.1 Perceived Social Support (Parents and close friends)

The subscales of perceived social support from parents and close friends from the Child and Adolescent Social Support Scale (CASSS; Malecki et al., 1999) had three items each and were answered on a 6-point Likert scale ranging from (1) *never* to (6) *always*. An example for perceived social support from parents was, “My parents … Listen to me when I need to talk” (T1, α = .81; T2. α = .84). An example for perceived social support from close friends was, “The friends I am with the most … Understand my feelings” (T1, α = .82; T2, α = .85).

#### 2.2.3 School adjustment

##### 2.2.3.1 Perceived social support (teacher)

The subscale of perceived social support from teachers from CASSS (Malecki et al., 1999) had five items, e.g., “My teachers … Care about me”, answered on the same 6-point Likert scale as above (T1, α = .81; T2, α = .84).

##### 2.2.3.2 School well-being

We asked participants two questions about their well-being in school, “I enjoy being in school” and “I feel safe at school”. The questions were answered on a 6-point Likert scale, ranging from (1) *never* to (6) *always* (T1, ρ = .77; T2, ρ = .83).

##### 2.2.3.3 Class well-being

We asked participants four questions about their well-being in class, examples are, “In my class we help one another” and “In my class we are kind to one another” (Skoog & Kapetanovic, 2020) (T1, α = .69; T2, α = .75). The items were answered on the same 6-point Likert scale used in previous stated measure.

### 2.3 Procedure

During the first time point, data were collected in the schools via questionnaires on the school computers or iPads. Researchers initially introduced the topic and the survey to the participants in person and were available on the premises to answer any questions the participants had. During the second time point, due to COVID-19 safety measures, the data were mostly collected online. Additionally, the students watched a pre-recorded video for the introduction to the survey and a live chat function was created for the students to be able to ask questions about the survey.

### 2.4 Statistical analysis

Statistical analyses were performed using R Statistical Software (version 4.0.3, R Core Team, 2020), using the packages ‘tidyverse’ and ‘rio’. Data missingness was inspected by using Little's MCAR test, a non-significant *p*-value was obtained indicating that data were missing completely at random (MCAR), X^2^ = 1471.89, df = 1477, *p* = .532. An index variable was made for each psychosocial factor for paired sample *t*-tests. Visual inspections of variable distributions, as well as Shapiro-Wilk tests, indicated violations of the assumption of normality. Despite the non-normality of the data, we chose to use parametric paired-sample *t*-tests, as there is evidence that most parametric tests are robust enough to withstand non-normal distributions (Knief & Forstmeier, 2021), and the interpretation of mean-level data is important to the study's research questions. To account for multiple tests and large sample size, we applied Bonferroni corrections.

## 3 Results

Descriptive statistics for the study variables and the paired sample *t*-tests along with Cohen's *d* effect sizes and confidence intervals are provided in Table 1. The paired *t*-tests showed significant mean-level decreases in almost all of students' psychosocial factors from T1 to T2. However, the effect sizes ranged from negligible to small according to Cohen's *d* (1988) standards. The largest mean-level decreases were found in students' perceived support from teachers (*M* difference = -.28), class and school well-being (*M* difference = -.25 and -.24, respectively), and students' self-esteem (*M* difference = -.31). There were no significant differences in students' emotional problems from T1 to T2. Overall, mean-level decreases were present in students' psychosocial well-being during the COVID-19 pandemic. The largest mean-level differences were found in students' school adjustment, in perceived support from teachers, school and class well-being, and in students' psychological adjustment in their self-esteem. These differences, however, had small effect sizes. Notably in students' psychological adjustments, students showed no differences in their emotional problems, and negligible differences in their sense of hope and self-efficacy.

**Table 1:** Descriptive statistics for variable indices, and paired t-tests across time along with effect sizes.

	Time 1			Time 2					Difference between T1 and T2	
Variables	Mean *(SD)*	Girls^†^	Boys ^††^	Mean *(SD)*	Girls ^†^	Boys^††^	Min	Max	Paired *t*-tests	Cohen's *d* (lower-upper CI)
Hope	4.77(*.89*)	4.71	4.83	4.61(*.96*)	4.46	4.80	1.00	6.00	−4.53(711) ***	.17 (.09;.25)
Self-efficacy	72.62(*22.95*)	69.03	76.94	69.75(*23.85*)	64.24	76.95	1.00	100	−3.61(754) **	.13 (.05;.20)
Self-esteem	4.13(*.93*)	4.06	4.18	3.82(*1.06*)	3.63	4.08	1.00	5.00	−6.75(805) ***	.27 (.18;.35)
Emotional problems	1.50(*.45*)	1.55	1.44	1.53(*.46*)	1.64	1.40	1.00	3.00	1.95(798)	
Support from friends	5.22(*.97*)	5.26	5.19	5.12(*1.02*)	5.10	5.13	1.00	6.00	−3.09(815) *	.12 (.04;.19)
Support from parents	5.63(.*69*)	5.65	5.59	5.53(*.78*)	5.47	5.61	1.00	6.00	−2.88(833) *	.12 (.03;.18)
Support from teachers	5.32(*.84*)	5.29	5.34	5.04(*.97*)	5.02	5.06	1.00	6.00	−7.84(809) ***	.29 (.21;.36)
School well-being	5.17(*.94*)	5.11	5.27	4.93(*.95*)	4.80	5.08	1.00	6.00	−7.74(836) ***	.26 (.19;.33)
Class well-being	4.90(*.80*)	4.86	4.95	4.65(*.86*)	4.59	4.72	1.00	6.00	−7.24(796) ***	.26 (.19;.34)

Note: *p < .05, **p < .01, ***p < .001, ^†^*N* = 440, ^††^*N* = 403. T1 data collection lasted from 10/2019 to 01/2020, T2 data collection lasted from 11/2020 to 02/2021.

## 4 Discussion

In this study, we aimed – for the first time known – to find out whether there were differences between middle school students' psychosocial well-being prior to and during the COVID-19 pandemic whilst attending school in Sweden. We compared students’ psychosocial factors of school adjustment, quality of connections to significant others and psychological adjustment prior to the pandemic (T1) to during the pandemic (T2). In sum, 8–10 months into the pandemic, students had slightly lower self-esteem compared to before the pandemic, felt slightly less supported by their teachers, and felt slightly less well at their schools and classrooms, but showed no differences in their experiences of emotional problems.

These findings need be understood in relation to the context of students' developmental stage and the COVID-19 pandemic. Middle school students in Sweden are in the developmental stage of late childhood/preadolescence. This is a sensitive stage, where many changes, e.g., hormonal, emotional, cognitive, and social occur (Bacter et al., 2021). Typical mean-level differences in psychosocial well-being (Meeus, 2016), driven by children's sensitivity to their environment (DelGiudice, 2018), can be expected as they develop. Despite schools up to grade six remaining open, there were several changes in Swedish students' school environment. First, there was higher absenteeism from both students and teachers (Ahlström et al., 2020). Second, teachers reported an increased workload due to high absenteeism during the pandemic (Lärarnas riksförbund, 2020). In addition, there was a noticeable employee turnover of the teachers and principals in the schools participating in PRISE. Lastly, there was a decrease in employed primary and middle school teachers from the school years of 2019/2020 to 2020/2021 (Statista, 2021). Thus, the differences found in students' school adjustment are plausibly attributed to the disruptions they had experienced over the course of the year.

It is possible that the notable changes in students' school environment could be linked to the differences found in student's self-esteem. Orth et al. (2018) conducted a meta-analysis addressing self-esteem across the lifespan. They found that self-esteem increased up to the age of 11, then remained constant until the age of 15. Fluctuations in self-esteem, however, are dependent on external feedback (Orth & Robins, 2019). Thus, the students from the current study may show slightly lower self-esteem due to disruptions in their environment. Although overall, student's psychological adjustment does not seem to be negatively affected by the pandemic, given that decreases in self-esteem were not equivalent to students having a characteristic of low self-esteem, and the differences in student's hope and self-efficacy have negligible effect sizes (Cohen, 1988). Support for students not being negatively affected is further given by the data showing no differences in emotional problems.

The absence of differences in emotional problems contrasts most international literature. This discrepancy may be due to international policy differences in approaches to the COVID-19 pandemic. Most countries that reported poorer mental well-being amongst children and adolescents, had strict lockdowns, curfews, and school closures. Creswell et al. (2021) found that when UK students were allowed back into school, their mental well-being improved in comparison to being in strict lockdown. Sweden has had a more open approach, allowing for some ‘normalcy’. Chen et al. (2021) argued that this allowance of ‘normalcy’ may be what has allowed for Swedish adolescents' psychosocial development to not have been as negatively impacted. Our study supports Chen's argument, showing a similar trend in Swedish children.

Finally, despite concerns about children's relationships with parents and close friends due to stress and social distancing during the pandemic (Masten & Motti-Stefanidi, 2020), Swedish children's relationships to significant others showed negligible differences in this study.

This study gives support to prior findings that suggest children's well-being return to similar pre-pandemic levels as time goes on (Robinson et al., 2021). We argue that the possibility of minor to no mean-level differences in psychosocial well-being is associated to positive adaptation. Previous literature show that the presence of protective factors, such as the psychosocial factors, and absence of maladjusted outcomes (e.g., internalizing symptoms) amidst the experience of adversity, are indicative of positive adaptation (Luthar & Cicchetti, 2000). Having multiple protective psychosocial factors has long-term implications for protecting children from multiple risk factors (Werner, 2013). Even if students' school environment may be less stable, other contexts may be able to compensate for that lack. In the current context of the COVID-19 pandemic, if students feel supported by their parent(s) or friends, and they also feel hopeful and believe in their own abilities, they can adapt positively, despite potential challenges they are facing within their school context.

### 4.1 Limitations & strengths

The foremost limitation of this study is that we were not able to directly measure the participants' perception and experience of the COVID-19 pandemic. We cannot determine direct effects of the pandemic on psychosocial well-being. Presumably, this pandemic has changed everyone's day-to-day lives. Furthermore, we relate the study findings to students' developmental context, and by that we can infer what may be driving some of the found mean-level differences within the context of the pandemic. Although, such underlying processes must be further explored in future research. It is a strength of the study to have been able to measure students' protective factors and emotional problems prior to the pandemic and make comparisons during the pandemic.

## 5 Conclusion

Children are in many ways a vulnerable group. Our study demonstrates general positive adaptation during the COVID-19 pandemic among Swedish middle school students. Students' emotional problems showed no differences, whilst small differences in student's relationships to significant others and factors of psychological adjustment may be partially due to typical mean-level changes during development (Meeus, 2016). Meaningful differences in students' school adjustment are plausibly attributed to disruptions caused by the pandemic. Holistically, students do not seem to be doing poorly. This study together with Chen et al. (2021) has shown that when students continue attending school, their psychosocial well-being does not worsen as it does for students experiencing school closures (Cresswell et al., 2021; Viner et al., 2021). This study is a steppingstone to understanding the development of student's resilience during the COVID-19 pandemic, which will be explored further in future research.

## Ethics

The PRISE study was approved by the Swedish Ethical Review Authority (reference number 2019–02755). The students’ legal guardians gave written informed consent. Students were orally informed about the research project, in a way as to facilitate understanding, and were informed of their right of withdrawal. The students gave oral informed consent on participating days.

## CRediT authorship contribution statement

**Emily G. Vira:** Conceptualization, Formal analysis, Methodology, Writing – original draft, Visualization. **Therése Skoog:** Conceptualization, Funding acquisition, Investigation, Methodology, Project administration, Resources, Supervision, Validation, Visualization, Writing – review & editing.

## Declarations of competing interest

None.
